# Academic Motivation of Indonesian University Students: Relationship with Self-Compassion and Resilience

**DOI:** 10.3390/healthcare10102092

**Published:** 2022-10-20

**Authors:** Yasuhiro Kotera, Muhammad Aledeh, Kristian Barnes, Annabel Rushforth, Habib Adam, Riswani Riswani

**Affiliations:** 1 School of Health Sciences, University of Nottingham, Nottingham NG7 2HA, UK; 2 Danube City Hospital, Vienna Health Association, Langobardenstraße 122, 1220 Vienna, Austria; 3 Moriarty, Flynn and Barnes, 51 Goldhill Plaza, #07-10/11, Singapore 308900, Singapore; 4 College of Health, Psychology and Social Care, University of Derby, Derby DE22 1GB, UK; 5 Department of Computer Science, University of Applied Sciences, FH Technikum Vienna, Höchstädtpl. 6, 1200 Wien, Austria; 6 Faculty of Education and Teacher Training, State Islamic University of Sultan Syarif Kasim Riau, Pekanbaru 28293, Indonesia

**Keywords:** Indonesian students, intrinsic motivation, extrinsic motivation, amotivation, self-compassion, resilience

## Abstract

Academic motivation is an important construct for university students, associated with student wellbeing and academic performance. Students who are motivated tend to feel and perform well. Self-compassion, that is kindness and understanding towards oneself in difficult times, and resilience, an ability to bounce back from difficulties, are also associated with student wellbeing and academic achievement. However, how these variables are related to each other has not been evaluated in Indonesian university students. Indonesian higher education has rapidly developed, focusing on student achievement while their wellbeing suffers. Understanding how academic motivation is linked with self-compassion and resilience can inform an effective way to augment their motivation. Accordingly, this cross-sectional study evaluated the relationship among these three variables. An opportunity sample of 156 students in Indonesia completed measures about those three constructs. Correlation, regression and moderation analyses were used. Intrinsic motivation was positively associated with extrinsic motivation and resilience. Amotivation was negatively associated with self-compassion and resilience. Self-compassion was positively associated with resilience. Both self-compassion and resilience predicted all three types of motivation apart from self-compassion for intrinsic motivation. Lastly, self-compassion nor resilience moderated the pathway from extrinsic motivation to intrinsic motivation. Resilience interventions are recommended for Indonesian students to maintain intrinsic motivation. Our findings will help educators and wellbeing staff to identify helpful ways to support healthful motivation in this student population that is undergoing drastic changes.

## 1. Introduction

### 1.1. Indonesian Universities’ Growth and Changes

The Indonesian government has been reforming its education policies to support its growing economy [1,2]. This includes mandates, such as the requirement for 20% of higher education (HE) students to be enrolled from lower socio-economic backgrounds [2]. The number of Indonesians attending HE has grown rapidly, from 17.23% of the population in 2005 to 36.31% in 2018 [2]. Additionally, the scholarly output from HE in Indonesia increased from 2500 articles in 2000 to over 45,000 in 2020, due to governmental focus and incentives [3]. The Indonesian governments’ continued investment in HE will ultimately contribute to a growth in work productivity, increased income and wealth, and overall wellbeing in the general population [4].

At the same time, there has been an increase in the number of students claiming poor mental wellbeing [5]. Several studies have shown the mental wellbeing of Indonesian higher education students is lower than those in other countries. Of Indonesian HE students, between 37–53% have reported high stress symptoms, with 51% feeling anxiety and 25% experiencing depression [6]. In Indonesia, there is both a lack of HE mental health services and an underutilisation of the existing services [7,8]. Effective approaches for improving their mental wellbeing are needed.

### 1.2. Academic Motivation, Self-Compassion and Resilience

Motivation is the process that determines the initiation, intensity and persistence of behaviours required to realise an individual’s defined goal or need [9]. Motivated behaviours are considered as energetic and focused [10] and motivation explains why an individual behaves in specific ways [11]. Academic motivation is a multivariate set of beliefs and behaviours, which are linked to students’ academic achievement and wellbeing, such as self-efficacy, self-regulation capability, learning engagement, perceived learning value, reasons for studying, achievement motives, goals, resilience and adaptability [12]. Research indicates the stronger a students’ academic motivation, the stronger the perception of their wellbeing is, and vice versa [13].

Self-determination theory (SDT) [14] explains academic motivation as a cognitive and behavioural drive to achieve academic success. SDT proposes motivation can be intrinsic (internal self-satisfying and fulfilling drivers), extrinsic (external drivers, such as parental pressure or high grades) or amotivation (no motivation) [14]. These are not exclusive motivational states but exist on a self-determined continuum, with intrinsic motivation being more autonomous and positive [15]. Intrinsically motivated students display higher subjective wellbeing [16], and achieve a higher academic performance [17]. On the other hand, extrinsically motivated students demonstrate higher levels of burnout, cheating, and dropout rates and a poorer academic achievement [18]. Extrinsically motivated students often focus on the external consequence (e.g., education as a steppingstone for better employment), therefore, seeing academic activities as obligatory [19]. With amotivation, a state where a student is simply unmotivated relates to poor mental health [18] and high drop-out rates [20]. Overall research in academic motivation reports intrinsic motivation is linked with better student wellbeing, whereas extrinsic motivation and amotivation are relevant to poor student wellbeing.

Self-compassion, kindness towards oneself, is also related to wellbeing. Self-compassion comprises three parts: being kind to oneself in difficult times, accepting failure and hardship are universal human experiences, and managing negative thoughts and feelings [21,22]. Student research has consistently reported self-compassion is associated with wellbeing and motivation [23,24,25,26]. Further, a study of UK students [12] showed self-compassion moderated the pathway from extrinsic to intrinsic motivation by strengthening that pathway, emphasising the importance of self-compassion on motivation to learn. Intrinsically motivated individuals tend to have higher levels of self-compassion and better mental wellbeing [27]. Additionally, students who practice self-compassion are less affected by negative feedback and suffer less stress and depressive symptoms [28,29,30]. Self-compassion is, therefore, important for students’ mental wellbeing and academic motivation [12,31,32].

Another key wellbeing construct is resilience. Resilience is generally defined as being able to marshal the necessary internal resources to overcome adversity and challenges [33]. Practicing resilience can strengthen the individual’s personality and psychological coping mechanisms [34]. Resilience theory argues it is how we cope in difficult situations [35], which matters more to wellbeing [36]. Research shows that self-compassion protects mental wellbeing by supporting resilience, suggesting self-compassion is a mediator between resilience and mental wellbeing [37]. Moreover, resilience and self-compassion are strongly related, suggesting that in student populations resilience interventions can cultivate self-compassion [23]. The ability to bounce back from academic setbacks is critical to students’ wellbeing, and self-compassion strengthens the impact of the resilience on wellbeing [32].

Gilbert’s three emotion regulatory systems is the theoretical background of this research [38]. This model posits that we have three emotion systems: threat, drive and soothing systems. The threat system is activated with fear and anxiety (e.g., the Fight–Flight–Freeze response). Emotions, such as fear and anxiety, are stressful, therefore, accessing this system for a long time leads to poor mental health. The drive system is incentive based and motivated towards an external reward. Although this system is often initiated by the temporary pleasure of getting the reward, we do not always get what we want in our life. When we do not get what we want, we feel depressed and self-critical. This also damages our mental health. Contrarily, the soothing is activated with safety and contentment. In the soothing system, we feel content with how things are. Compassion is expressed when we engage with the soothing system [38]. Recent studies identified that intrinsic motivation is more closely linked with self-compassion than extrinsic motivation [12]. An intrinsically motivated person is aware of their own passion and interest, which are more pertinent to emotions in the soothing system (e.g., safety, contentment) [34]. In a UK sample, self-compassion strengthened a pathway from extrinsic motivation to intrinsic motivation [12]. This partially helps explain why intrinsic motivation is associated with mental wellbeing. Likewise, resilience is also a positive wellbeing construct, closely related to self-compassion [39]. Resilience requires self-awareness. Being aware of our own emotions and values (instead of being reactive or judgmental) can soothe ourselves (the soothing system). In another UK sample, among various positive wellbeing constructs, resilience was most closely associated with self-compassion [23]. Using the three emotion regulatory systems, the healthful constructs of self-compassion and resilience should support a pathway from extrinsic motivation to intrinsic motivation, activating the soothing system. Stronger self-compassion and resilience would facilitate the pathway. However, this has not been investigated in Indonesian students.

In sum, intrinsic motivation, self-compassion and resilience are wellbeing constructs, associated with good mental health in many populations [40]. Moreover, how self-compassion and resilience can assist students’ intrinsic motivation—a key construct for mental wellbeing—remains to be assessed in this population. Identifying these relationships would be meaningful because Indonesian students undergo rapid changes, which challenge their mental health (as seen in the recent pandemic [41,42]). Findings from this study can help identify effective approaches for protecting the mental health in this student group.

### 1.3. Study Aims

The aim of this study was to assess the academic motivation of Indonesian university students. Firstly, the relationships among academic motivation (i.e., intrinsic motivation, extrinsic motivation and amotivation), self-compassion and resilience were examined (Aim 1). Secondly, whether self-compassion and resilience independently predicted each type of academic motivation was explored (Aim 2). Lastly, whether self-compassion and resilience moderated the extrinsic–intrinsic motivation pathway was evaluated (Aim 3).

## 2. Materials and Methods

The study design was cross-sectional. This design helps to identify determinants of mental health outcomes, and allows timely output [43].

### 2.1. Participants

The participants constituted an opportunity sample of 156 students at an Indonesian university who completed measures about three constructs. They were recruited voluntarily: none of the participants received any reward for their participation. The inclusion criteria were being 18 years old or above and studying a caring profession subject (e.g., counselling and education) full-time. Students who were taking a study break from their studies were excluded from this study.

The study materials comprised the study information sheet, consent form, survey and debriefing, and were prepared as hard copies. These were disseminated by an independent tutor to 162 students between October to December 2021. There was a 96% (*n* = 156) response rate. The sample size satisfied the required size calculated by power analysis (84: two tails, *p*H1 (*r*) = 0.30 (medium [44]), *α* = 0.05, Power = 0.80, *p*H0 = 0 [45]). The respondents were majority female (*n* = 128) while 25 of them were males and 3 of them did not disclose their gender. The age range was 18–22 years (19.07 ± 0.98), and there were five postgraduate students among the participants. The study sample was similar in age and gender balance to the general Indonesian student population which was female-dominated (Age: 20 years old, 49% [46]).

### 2.2. Ethical Approval

The study was approved by the ethics committee of the State Islamic University of Sultan Syarif Kasim Riau: Ref KE/KEP-FPP/01/05/2022, and there were no financial incentives involved.

### 2.3. Materials

Three self-report scales, prepared in English, were used.

Academic motivation was assessed using the Academic Motivation Scale (AMS) [47]. AMS, a 28-item scale, considers the three types of motivation introduced above: amotivation, extrinsic motivation and intrinsic motivation. Example items are ‘I once had good reasons for going to college; however, now I wonder whether I should continue’ for amotivation; ‘Because with only a high-school degree I would not find a high-paying job later on’ for extrinsic motivation’, and ‘For the pleasure that I experience in broadening my knowledge about subjects which appeal to me’ for intrinsic motivation. All items are answered on a seven-point Likert scale (from 1 = ‘Does not correspond at all’ to 7 = ‘Corresponds exactly’). All of the subscales have adequate to high reliability with Cronbach’s alphas between 0.62 and 0.91 [48].

The Self-Compassion Scale-Short Form (SCS-SF), a brief version of the original 26-item Self-Compassion Scale, was used [49]. There are 12 items in SCS-SF, answered on a five-point Likert scale (e.g., ‘I try to see my failings as part of the human condition’; 1 = ‘Almost never’ to 5 = ‘Almost always’; Responses for the items 1, 4, 8, 9, 11 and 12 are reversed). Cronbach’s alpha was high (0.86) [49].

Resilience was measured using the Brief Resilience Scale (BRS; six items) [50]. Example items include ‘I tend to bounce back quickly after hard times’ and are responded on a five-point Likert scale (1 = ‘Strongly disagree’ to 5 = ‘Strongly agree’; Responses for the items 2, 4 and 6 are reversed). BRS had high internal consistency (α = 0.80–0.91; [50]).

### 2.4. Analysis

SPSS (v28) and Process Macro version 3 were used. After screening for parametric tests, correlation analysis and multiple regression analysis were performed to address Aims 1 and 2. Lastly, moderation analysis was conducted to assess whether self-compassion or resilience moderates the pathway from extrinsic motivation to intrinsic motivation (Aim 3).

## 3. Results

The results were categorised to report the outcome of each aim.

### 3.1. Correlations among Academic Motivation, Self-Compassion and Resilience (Aim 1)

Point biserial correlations were used for gender (1 = male, 2 = female). Table 1 summarises the results of our correlation analysis. Age was negatively associated with resilience. Intrinsic motivation was positively associated with extrinsic motivation and resilience. Amotivation was negatively associated with self-compassion and resilience. Self-compassion was positively associated with resilience.

### 3.2. Predicting Academic Motivation (Aim 2)

To further examine the relationships among each type of academic motivation, self-compassion and resilience, multiple regression analyses were conducted. Each type of academic motivation (intrinsic motivation, extrinsic motivation and amotivation) was entered as an outcome variable, and self-compassion and resilience were entered as predictor variables (Table 2). Multicollinearity was of no concern (variance inflation factors < 10).

Self-compassion and resilience accounted for 3–7% of each type of academic motivation (small effect sizes [44]), where amotivation was predicted to the largest degree (7%). Both self-compassion and resilience were identified as significant predictors of all three types of academic motivation (*p* < 0.05), apart from self-compassion’s prediction of intrinsic motivation (*p* = 0.34).

### 3.3. Moderation of Extrinsic-Intrinsic Motivation Pathway (Aim 3)

**Figure 1:**
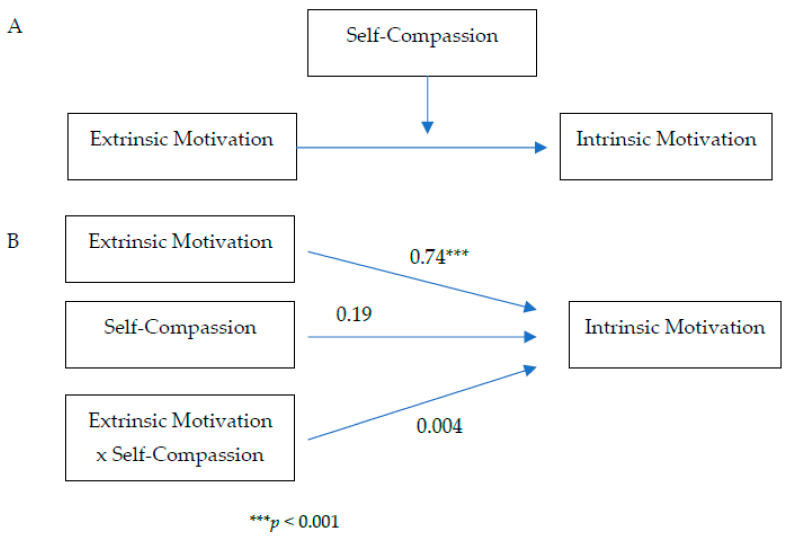
Moderation of self-compassion on the pathway from extrinsic motivation to intrinsic motivation: conceptual diagram (**Panel A**) and statistical diagram (**Panel B**).

**Figure 2:**
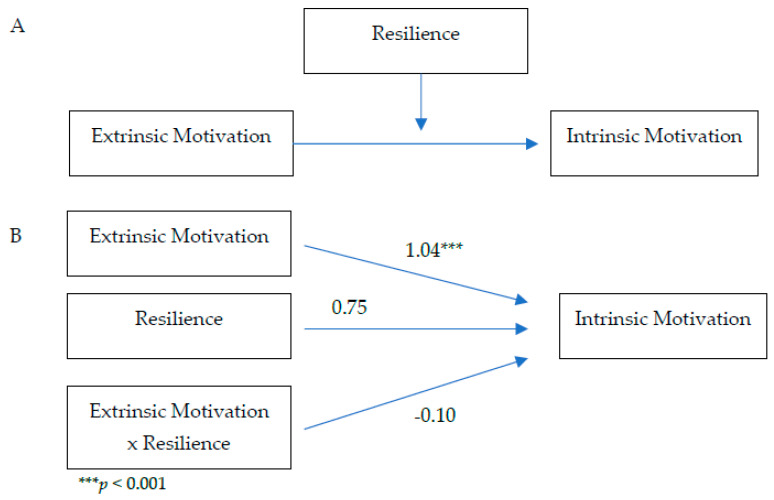
Moderation of resilience on the pathway from extrinsic motivation to intrinsic motivation: conceptual diagram (**Panel A**) and statistical diagram (**Panel B**).

Whether self-compassion or resilience moderates the pathway from extrinsic motivation to intrinsic motivation was evaluated using Model 4 in the Process macro (parallel mediation model [51]) (see Figure 1 and Figure 2).

Neither self-compassion nor resilience moderated the pathway from extrinsic motivation to intrinsic motivation as indicated by the non-significant interaction effects: *b* = 0.004, *p* = 0.95 for extrinsic motivation and self-compassion, *b* = −0.10, *p* = 0.28 for extrinsic motivation and resilience.

## 4. Discussion

This study evaluated the academic motivation of Indonesian university students, and its relationships with self-compassion and resilience. Firstly, our analysis revealed that intrinsic motivation was positively associated with extrinsic motivation and resilience while amotivation was negatively associated with self-compassion and resilience. Furthermore, self-compassion was positively associated with resilience. Significant relationship between intrinsic motivation and extrinsic motivation was found consistently in other student populations [21,52,53]. This may mean that in students’ intrinsic motivation and extrinsic motivation are not mutually exclusive experiences. This is in line with organismic integration theory, a theory embedded in SDT, which posits that extrinsic motivation contributes to the development of intrinsic motivation [54,55]. The positive association between intrinsic motivation and resilience suggests Indonesian students who are passionate about and more connected to their studies tend to be more resilient. This is in line with previous research showing a positive significant relationship between intrinsic motivation and resilience in American students, implying that students that are intrinsically driven to achieve their educational goals are more likely to be equipped with the resilience mechanisms that allow them to overcome the difficulties associated with educational challenges [56]. Extrinsic motivation saw no significant relationship with either self-compassion or resilience. This is surprising given the strong associations seen between extrinsic motivation and reduced mental health [57,58], suggesting that this relationship is not affected by these constructs. Additionally, amotivation was negatively correlated with both self-compassion and resilience. This implies that levels of self-compassion and resilience are not salient in the Indonesian student population regarding their motivation.

Secondly, our results showed through regression analysis that both self-compassion and resilience were predictors for extrinsic motivation and amotivation, and resilience was a predictor for intrinsic motivation. Self-compassion did not significantly predict intrinsic motivation. This differs from previous research showing that students who practice self-compassion have greater motivation to learn [26,27]. This may be because in collectivist cultures, wellbeing and stress are experienced differently than in western cultures [59,60]. This may be due to psychological attributes characterising the self, such as self-esteem, being less relevant to the wellbeing of the individual in collectivist cultures, as the goals, and thus the motivation of these goals, of the group tend to take priority over the goals of the individual [61]. Additionally, in individualistic cultures, taking care of oneself promotes awareness of themselves, including their passion (i.e., intrinsic motivation), whereas in collectivist culture, self-compassion is more about the sameness with others (e.g., common humanity) and, therefore, is less relevant to intrinsic motivation [48].

Neither self-compassion nor resilience moderated the pathway from extrinsic motivation to intrinsic motivation. This shows that the student level of self-compassion is not relevant with regard to becoming more intrinsically motivated. This is not in line with previous research in UK students, where self-criticism and self-compassion moderated the pathway from extrinsic motivation to intrinsic motivation, as higher self-criticism weakened the pathway and higher self-compassion strengthened it [12]. Self-compassion is conceptualised as self-kindness and self-acceptance in times of stress and perceived self-inadequacy [62]. As moderation of this pathway was not seen, this implies that perceived self-inadequacy may not be inhibiting intrinsic motivation. This again can be explained by the UK–Indonesia cultural difference. UK culture is highly individualistic, with an emphasis placed on personal achievement and independence [63]. Indonesian culture is more collectivist, and thus the emphasis is placed more on group achievement [64]. More individualistic and success-driven cultural values create a more competitive system, evoking social comparison [65], and thus self-compassion training would be effective in neutralising this. This may explain why perceived self-inadequacy and self-compassion is less saliant regarding intrinsic motivation in the Indonesian student population, as the self-judgment may be less impactful on an individual’s feelings, and thus motivation regarding their academic achievement. Additionally, self-compassion is understood to protect mental wellbeing by supporting resilience [23]. This implies that self-compassion and resilience interventions that are seen to be successful in affecting motivation in individualistic cultures may not be as effective in promoting intrinsic motivation in collectivist cultures. Future research into the components that do moderate the pathway from extrinsic motivation to intrinsic motivation in Indonesian students will be able to inform intervention selection for this population.

Taken together, results in our sample of Indonesian students were somewhat different from other populations in individualistic cultures. Intrinsic motivation and self-compassion were associated with each other in many other samples, but not in the Indonesian student sample. We suggest this may be due to collectivistic experience of self-compassion as opposed to individualistic experience of self-compassion, focusing on the self [66]. Future research is needed to understand which component of self-compassion is related the most to each type of motivation among Indonesian students.

In practice, Indonesian educators may want to introduce resilience to impact student motivation instead of self-compassion. Though it is still nascent, resilience training was offered to Indonesian students and improved their mental wellbeing [67]. One advantage of resilience may be that resilience has been incorporated in various types of training. For example, a martial arts programme was introduced to students and enhanced the level of their resilience [68,69]. Peer dialogue using questions about difficult emotions, an exercise named ‘emotional speed-dating’ was effective for enhancing student resilience [70]. Forest bathing, paying attention to the senses while spending time in a forest, also improves resilience [71,72,73]. Online resilience training is now actively developed and has shown positive effects [74,75]. These already-implemented approaches targeted resilience should be offered to Indonesian students.

## 5. Limitations

Several limitations to be noted. First, our sample was recruited using availability sampling at one university in Indonesia. This may limit the generalisability of our findings. Second, the use of self-report scales might have compromised the accuracy [76]. Relatedly, there has been a debate about the accuracy and reliability of the SCS-SF [77]. AMS comprised 28 items: the short 14-item version could have been used to reduce the workload of the students [78]. The causality of the variables was not evaluated in this study. Lastly, we could not conduct sub-scale analysis of self-compassion. This may be particularly useful, because the results differed between the UK samples and this Indonesian sample. Evaluating which component of self-compassion may explain the difference in this study could have been possible with the sub-scale scores. For example, common humanity, one component of self-compassion may be more in line with collective culture values, whereas self-kindness, another self-compassion component, may be more pertinent to individualistic culture values due to its ‘self’ nature. These component analysis across cultures has not been done in self-compassion research. Future research needs to examine these aspects of self-compassion.

## 6. Conclusions

Academic motivation is important, however, its relationship with key mental wellbeing constructs, self-compassion and resilience, remained to be evaluated in students in Indonesian higher education. Indonesian higher education has been going through substantial changes, and student wellbeing has suffered. Resilience was associated with intrinsic motivation, whereas self-compassion was not. Unlike western student samples, self-compassion was not relevant to intrinsic motivation in Indonesian students. Resilience training should be introduced to Indonesian students to maintain their academic motivation.

## Figures and Tables

**Table 1:** Correlation among each type of academic motivation (intrinsic motivation, extrinsic motivation and amotivation), self-compassion and resilience in 156 Indonesian students.

		M	SD	α	1	2	3	4	5	6	7
1	Gender (1 = M, 2 = F)	M(25), F(128), Unreported(3)			-						
2	Age	19.07	0.98		−0.12	-					
3	Intrinsic Motivation	4.66	1.11	0.91	−0.02	−0.04	-				
4	Extrinsic Motivation	5.10	1.25	0.92	0.004	−0.004	0.84 **	-			
5	Amotivation	2.88	1.40	0.77	0.02	−0.10	0.05	−0.11	-		
6	Self-Compassion	3.32	0.50	0.67	0.02	0.09	0.01	−0.10	−0.24 **	-	
7	Resilience	3.08	0.39	0.53	−0.004	−0.17 *	0.20 *	0.13	−0.24 **	0.39 **	-

* *p* < 0.05; ** *p* < 0.01 (two-tailed).

**Table 2:** Multiple regression: Self-compassion and resilience for each type of academic motivation (intrinsic motivation, extrinsic motivation and amotivation) in 156 Indonesian students.

	Outcome: Intrinsic Motivation				
	B	SE_B_	β	95%CI (L, U)	
Self-Compassion	−0.18	0.19	−0.08	−0.56	0.20
Resilience	0.65 **	0.24	0.23	0.17	1.13
Δ Adj. R^2^	0.03				
	Outcome: Extrinsic Motivation				
	B	SE_B_	β	95%CI (L, U)	
Self-Compassion	−0.44 *	0.22	−0.18	−0.87	−0.02
Resilience	0.63 *	0.27	0.20	0.09	1.17
Δ Adj. R^2^	0.04				
	Outcome: Amotivation				
	B	SE_B_	β	95%CI (L, U)	
Self-Compassion	−0.49 *	0.24	−0.17	−0.96	−0.02
Resilience	−0.63 *	0.30	−0.18	−1.23	−0.04
Δ Adj. R^2^	0.07				

* p < 0.05; ** *p* < 0.01.

## Data Availability

The data presented in this study are available on request from the corresponding author. The data are not publicly available due to ethical consideration.
